# Agomelatine ameliorates abortion‐related alterations in rats: Insights from behavioural, biochemical and histological analyses

**DOI:** 10.1113/EP093951

**Published:** 2026-10-05

**Authors:** Mehtap Pervin, Zeynep Dila Oz, Ahmed Sait Bozyil, Orhan Sayin, Husna Batmaz, Serhat Hancer, Salih Burcin Kavak, Nevin Kocaman, Haluk Kelestimur, Emine Kacar

**Affiliations:** ^1^ Department of Physiology, Faculty of Medicine Firat University Elazig Turkey; ^2^ Department of Histology & Embryology Gaziantep City Hospital Gaziantep Turkey; ^3^ Department of Obstetrics and Gynecology, Faculty of Medicine Firat University Elazig Turkey; ^4^ Department of Histology & Embryology, Faculty of Medicine Firat University Elazig Turkey; ^5^ Department of Physiology, Faculty of Medicine Istanbul Okan University Istanbul Turkey

**Keywords:** abortion, agomelatine, forced swimming test, mifepristone, rat

## Abstract

Abortion aetiology is critically linked to increased myometrial contractions and maternal psychological stress. Given the limited efficacy of current therapies such as progesterone, investigating novel therapeutic interventions is essential. Melatonin exerts reproductive benefits. Agomelatine stands out as an agent that exerts antidepressant effects through serotonin receptor (5‐HT_2C_) antagonism in addition to its potent melatonergic activity. Furthermore, agomelatine inhibits myometrial contractions. In this context, this study investigates agomelatine's therapeutic potential in an abortion model across behavioural, biochemical and histological parameters. Eighty female Sprague–Dawley rats were randomized into eight groups: control, sham, abortion, melatonin (M), agomelatine (A), progesterone (P) and combinations (MP, AP). Following pregnancy (Day 0), oral treatments were administered daily for 14 days. Abortion was induced via mifepristone on Day 11. Depressive‐like behaviours were assessed using a forced swimming test on Day 13. The next day, rats were sacrificed for serum hormone analysis and uterine histological evaluation. The forced swimming test showed shorter immobility time in the AP group compared to the abortion group. An enzyme‐linked immunosorbent Assay revealed lower serum prostaglandin F_2α_ level in the AP group and lower serum oxytocin levels in the M, A, MP and AP groups versus the abortion group. Histologically, M, A, P, MP and AP groups exhibited higher scores for complete fetus, decidual congestion, decidual inflammation and cytotrophoblast–syncytiotrophoblast cells compared to the abortion group. Additionally, the abortion group showed higher decidual necrosis than controls. In conclusion, agomelatine‐containing protocols demonstrated restorative effects in abortion across behavioural, biochemical and histological parameters. These effects of agomelatine provide a new perspective on the currently limited options for treatment of abortion.

## Abstract (highlights)

- **What is the central question of this study?**
  Does agomelatine ameliorate behavioural alterations, biochemical changes and histological damage induced by abortion?
- **What is the main finding and its importance?**
  Agomelatine ameliorates behavioural, biochemical and histological impairments induced by abortion through the reduction of depressive‐like behaviours, modulation of hormonal levels, and preservation of fetal and decidual structural integrity. By mitigating tissue damage and supporting pregnancy maintenance, this compound offers a novel therapeutic perspective for currently limited options in abortion management.

## 1 INTRODUCTION

Abortion is defined by the World Health Organization as the expulsion of all or part of an embryo or fetus and its appendages weighing less than 500 g from the uterus before the 20th week of gestation (Cunningham & Leveno, 2022) Approximately 30% of pregnancies terminate in abortion (Sezer et al., 2009). Chromosomal abnormalities, previous abortions, advanced maternal age, smoking, alcohol consumption and psychosocial stress factors are prominent in the aetiology (Quenby et al., 2021). Although bed rest and progesterone administration are widely utilized in the current management of abortion risk, progesterone appears insufficiently effective in preventing pregnancy loss (Aleman et al., 2005; Sotiriadis et al., 2004). Furthermore, even though progesterone remains the standard choice in managing abortion risk, high progesterone levels can be associated with psychological effects. Maternal psychological stress is also an independent risk factor for pregnancy loss, and these losses have social and societal consequences in addition to their physiological effects (Herbert et al., 2022). Particularly in pregnancies accompanied by psychosocial stress, developing multidimensional therapeutic strategies that can simultaneously preserve both reproductive physiology and psychological well‐being is of critical importance.

Agomelatine is a melatonergic antidepressant with a pregnancy category B rating, known to exert its effects through melatonin receptor type 1 (MT_1_) and melatonin receptor type 2 (MT_2_) agonism and 5‐hydroxytryptamine receptor 2C (5‐HT_2C_) antagonism (Kacar et al., 2023; Kennedy & Rizvi, 2010). The effects of melatonin on reproductive physiology have been established through various studies in the literature. It has been reported to support the development of the endometrium and uterine glands, as well as to increase fetal counts and implantation rates (Chuffa et al., 2020; Zhang et al., 2017). Although the inhibitory effect of agomelatine, a melatonergic antidepressant, on myometrial contractions has been demonstrated in experimental models, studies examining its systemic effects on reproductive physiology are quite limited (Kacar et al., 2023). Furthermore, the drug's potential to increase dopamine and noradrenaline levels through 5‐HT_2C_ antagonism has secondary protective potential against stress, which is one of the risk factors for abortion (Fornaro et al., 2010; Millan et al., 2003; Qu et al., 2017; Srinivasan et al., 2012). When agomelatine's potential impact on reproductive physiology via melatonergic activity and its antidepressant properties are considered together, it can be hypothesized that this agent may have a significant role in abortion treatment. The primary aim of this study is to investigate the potential therapeutic efficacy of agomelatine in an experimental abortion model from behavioural, biochemical and histological perspectives.

## 2 METHODS

### 2.1 Ethics approval

The investigators explicitly confirm their understanding of, and full compliance with, the animal ethical principles under which *Experimental Physiology* operates. This study complies with the journal's animal ethics policy, and all experimental procedures were conducted in accordance with the principles of the *Guide for the Care and Use of Laboratory Animals*. All animal experiments were conducted in accordance with institutional guidelines and were approved by the relevant ethics committee (Ethics No: 18.04.2023‐2023/07‐04). Permission was obtained from Firat University Experimental Research Center. The experimental room was maintained on a regulated 12‐h light/dark schedule (07.00–19.00 h). Throughout the entire experimental period, the animals had unrestricted access to food and water ad libitum. The rats were only mildly affected by the experimental procedures. At the end of the experiment, to prevent potential confounding effects of anaesthetic agents on the measured hormonal parameters, all experimental animals were humanely decapitated.

### 2.2 Animals and drug treatments

In this study, a total of 80 intact female Sprague–Dawley rats, weighing approximately 220 g and aged 8–10 weeks with regular oestrous cycles, were used (Marcondes et al., 2002). Female rats determined to exhibit regular cycles via a vaginal smear for 14 days were mated with an adult male during the oestrus phase. Rats in which sperm were detected in the vaginal smear at 09.00 h on the day following mating were considered pregnant, and these date were recorded as gestational day 0 (Xue et al., 2015). A total of eight experimental groups were established, with 10 animals in each group. All treatments were administered via oral gavage for 14 days, starting from gestational day 0 between 09.00–10.00 h (Figure 1).

**FIGURE 1:**
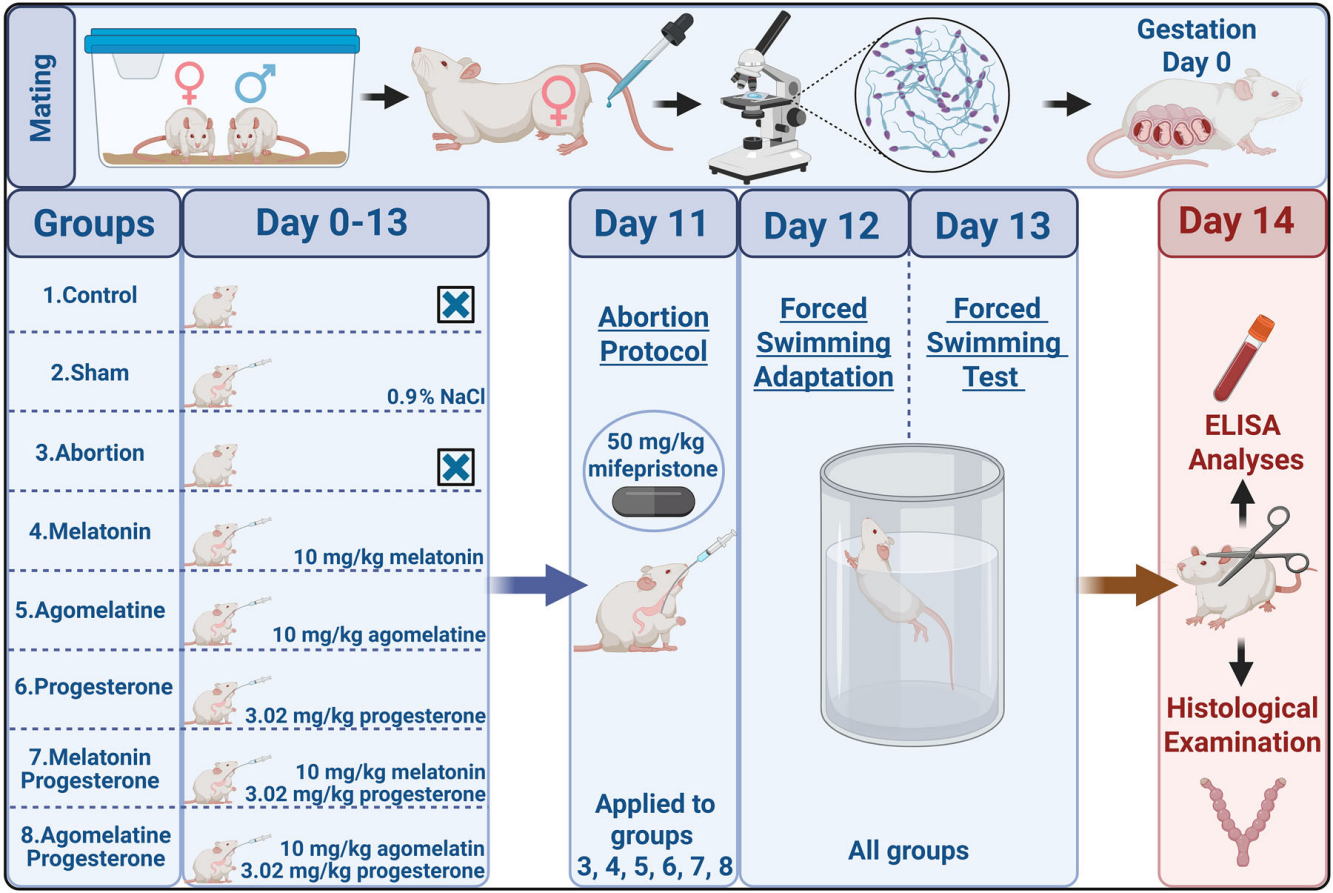
Flowchart of the experiment. Prepared using BioRender.com.

The eight experimental groups (Figure 1) were as follows:

1. Control: rats received no treatment.
2. Sham: saline (0.9% NaCl) was administered to the rats.
3. Abortion: rats received no treatment.
4. Melatonin (M): melatonin (10 mg/kg; Galenik Ecza, Izmir, Turkey, cat. no.: 73‐31‐4) dissolved in saline was administered (Arinno et al., 2021).
5. Agomelatine (A): agomelatine (10 mg/kg; Valdoxan®, Istanbul, Turkey) dissolved in saline was administered (Canpolat et al., 2016).
6. Progesterone (P): progesterone (3.02 mg/kg; Cayman Chemical Co., Ann Arbor, MI, USA, cat. no.: 15876) dissolved in sesame oil was administered (Dai et al., 2019).
7. Melatonin+progesterone (MP): melatonin (10 mg/kg) (Arinno et al., 2021) dissolved in saline, and progesterone (3.02 mg/kg) (Dai et al., 2019) dissolved in sesame oil were administered.
8. Agomelatine+progesterone (AP): agomelatine (10 mg/kg) (Canpolat et al., 2016) dissolved in saline, and progesterone (3.02 mg/kg) (Dai et al., 2019) dissolved in sesame oil were administered.

### 2.3 Experimental abortion model

In all rats except for the control and sham groups, an experimental abortion model was established on gestational day 11 by administering 50 mg/kg mifepristone (Cayman Chemical, cat. no.: 10006317; Ann Arbor, MI, USA) dissolved in 0.1% dimethyl sulfoxide + saline via oral gavage, subsequent to the daily administration of the respective agents (Camilleri & Sammut, 2023) (Figure 1). To confirm the abortion, weight loss was monitored by weighing the animals immediately before the application on the day of mifepristone administration (day 11) and 24 h later (day 12). Additionally, the presence of bright red blood stains around the vaginal area was monitored (Camilleri & Sammut, 2023).

### 2.4 Forced swimming test

To investigate the effects of the administered treatments on depressive‐like behaviour, all animals were subjected to a forced swimming test on gestational day 13 (Figure 1). One day prior to the test (day 12), rats were acclimated to the environment by swimming at a temperature of 25 ± 2°C in cylindrical Plexiglas test apparatus with a height of 60 cm, a diameter of 25 cm and a water depth of 35 cm (Alamri et al., 2023; Al‐Ramadhan et al., 2023; Rahati Quchani et al., 2023). On gestational day 13, the swimming test was performed for 5 min and video‐recorded for scoring. Scoring was calculated by the same observer as follows: total swimming time = mobility (climbing, swimming) + immobility (making only the movements necessary to keep the head above water).  Following each trial, the water was completely replaced to prevent any residual odours from influencing subsequent test results.

### 2.5 Blood and tissue collection

Blood samples and uterine tissues were collected following sacrifice on gestational day 14 (Figure 1). Blood samples were centrifuged at 2790 *g* for 10 min, and the resulting serum samples were stored at −20°C for enzyme‐linked immunosorbent assay (ELISA). Uterine tissues were photographed macroscopically and then placed in containers containing 10% formaldehyde solution for microscopic examination.

### 2.6 Hormone analyses

Serum levels of prostaglandin F2α (PGF_2α_), oxytocin, β‐human chorionic gonadotropin (β‐hCG), prolactin, oestrogen, progesterone were determined via ELISA. Hormone concentrations were established by measuring optical densities at a wavelength of 450 nm using an ELISA reader (Thermo Scientific, USA) in accordance with the protocols described in the kits. The following ELISA kits were utilized: rat PGF_2α_ (PDGF2A) ELISA kit (USCN CEA749GE; Wuhan, China, detection range: 9.88–800 pg/mL, sensitivity: 4.13 pg/mL), rat oxytocin (OT) ELISA kit (SunRedBio 201‐11‐1725; Shanghai, China, detection range: 2–600 ng/L, sensitivity: 1.672 ng/L), rat β‐hCG ELISA kit (SunRedBio 201‐11‐6681; detection range: 1.5–400 mIU/mL, sensitivity: 1.302 mIU/mL), rat prolactin (PRL) ELISA kit (SunRedBio 201‐11‐0191; detection range: 1–200 ng/mL, sensitivity: 0.544 ng/mL), rat oestrogen (E) ELISA kit (SunRedBio 201‐11‐0177; detection range: 1–300 ng/L, sensitivity: 0.752 ng/L) and rat progesterone (PROG) ELISA kit (SunRedBio 201‐11‐0742; detection range: 2–300 ng/mL, sensitivity: 1.115 ng/mL).

### 2.7 Histological examination

Histological analyses were performed by a blinded histologist using a histoscore method (0: none, 1: mild, 2: moderate, 3: severe). Macroscopic images of uterine tissues obtained following sacrifice were scored based on three criteria: ‘complete fetus’, ‘fetal remains (partial abortion)’ and ‘bamboo‐like appearance (complete abortion)’ (Table 1; Figure 4). For microscopic examination, uterine tissues were stained with haematoxylin–eosin, and intrauterine residual cells were evaluated under a light microscope based on five distinct criteria: decidual congestion, decidual inflammation, decidual necrosis, cytotrophoblast cells and syncytiotrophoblast cells (Erdoğan et al., 2025; Zhang et al., 2016) (Table 2; Figures 5, 6, 7, 8).

**TABLE 1:** Statistical analysis of histoscore data obtained from macroscopic images of uterine tissues

	Complete fetus	Fetal remains	Bamboo‐like appearance
Control	3 ± 0^a^	0 ± 0^a^	0 ± 0^a^
Sham	2.9 ± 0.32^a^	0 ± 0^a^	0 ± 0^a^
Abortion	0 ± 0^b^	2.9 ± 0.32^b^	3 ± 0^b^
M	2.5 ± 0.71^a^	0.7 ± 1.06^a^	0.8 ± 1.14^a^
A	2.6 ± 0.70^a^	0.3 ± 0.67^a^	0.6 ± 1.08^a^
P	2.8 ± 0.42^a^	0.4 ± 0.70^a^	0.5 ± 0.85^a^
MP	2.7 ± 0.48^a^	0.2 ± 0.42^a^	0.3 ± 0.67^a^
AP	2.9 ± 0.32^a^	0.1 ± 0.32^a^	0.1 ± 0.32^a^

*Note*: Data are presented as means ± standard deviation; *n* = 10 per group. Statistical analyses were performed using the Kruskal–Wallis test followed by Dunn's *post hoc* test. Histological evaluation data were compared between groups (rows). Groups sharing at least one common letter do not differ significantly, whereas groups not sharing a common letter are statistically significantly different: complete fetus score compared to abortion group: control, *P *< 0.0001; sham, *P *< 0.0001; M, *P* = 0.003; A, *P* = 0.001; P, *P *< 0.0001; MP, *P* = 0.0003; AP, *P *< 0.0001; fetal remains score compared to abortion group: control, *P *< 0.0001; sham, *P *< 0.0001; M, *P* = 0.006; A, *P *< 0.0001; P, *P* = 0.001; MP, *P *< 0.0001; AP, *P *< 0.0001; bamboo‐like appearance scores compared to abortion group: control, *P *< 0.0001; sham, *P *< 0.0001; M, *P* = 0.006; A, *P* = 0.001; P, *P* = 0.001; MP, *P *< 0.0001; AP, *P *< 0.0001.

Abbreviations: A, agomelatine; AP, agomelatine + progesterone; M, melatonin; MP, melatonin + progesterone; P, progesterone.

**TABLE 2:** Statistical analysis of histoscore data regarding decidual changes.

	Decidual congestion	Decidual inflammation	Decidual necrosis	Cytotrophoblast	Syncytiotrophoblast
Control	2.4 ± 0.52^a^	2.7 ± 0.48^a^	0.4 ± 0.52^a^	2.8 ± 0.42^a^	2.9 ± 0.32^a^
Sham	2.5 ± 0.53^a^	2.5 ± 0.53^a^	0.5 ± 0.53^a^	2.9 ± 0.32^a^	2.8 ± 0.42^a^
Abortion	0.7 ± 0.48^b^	0.4 ± 0.52^b^	2.2 ± 1.03^b^	0.1 ± 0.32^b^	0.2 ± 0.42^b^
M	2.6 ± 0.52^a^	2.5 ± 0.53^a^	1.2 ± 1.03^ab^	2.5 ± 0.53^a^	2.4 ± 0.52^a^
A	2.7 ± 0.48^a^	2.6 ± 0.52^a^	1.6 ± 1.17^ab^	2.8 ± 0.42^a^	2.7 ± 0.48^a^
P	2.8 ± 0.42^a^	2.7 ± 0.48^a^	1.3 ± 1.16^ab^	2.6 ± 0.52^a^	2.7 ± 0.48^a^
MP	2.6 ± 0.52^a^	2.8 ± 0.42^a^	0.9 ± 1.10^ab^	2.8 ± 0.42^a^	2.6 ± 0.52^a^
AP	2.3 ± 0.48^a^	2.8 ± 0.42^a^	1 ± 1.05^ab^	2.7 ± 0.48^a^	2.8 ± 0.42^a^

*Note*: Data are presented as mean ± standard deviations; *n* = 10 per group. Statistical analyses were performed using the Kruskal–Wallis test followed by Dunn's *post hoc* test. Histological evaluation data were compared between groups (rows). Groups sharing at least one common letter do not differ significantly, whereas groups not sharing a common letter are statistically significantly different: decidual congestion compared to abortion group: control, *P* = 0.007; sham, *P* = 0.002; M, *P* = 0.0003; A, *P *< 0.0001; P, *P *< 0.0001; MP, *P* = 0.0003; AP, *P* = 0.029; decidual inflammation compared to abortion group: control, *P* = 0.0002; sham, *P* = 0.005; M, *P* = 0.005; A, *P* = 0.001; P, *P* = 0.0002; MP, *P *< 0.0001; AP, *P *< 0.0001; decidual necrosis compared to abortion group: control, *P* = 0.011; sham, *P* = 0.030; cytotrophoblast cells compared to abortion group: control, *P *< 0.0001; sham, *P *< 0.0001; M, *P* = 0.009; A, *P *< 0.0001; P, *P* = 0.002; MP, *P *< 0.0001; AP, *P* = 0.0003; syncytiotrophoblast cells compared to abortion group: control, *P *< 0.0001; sham, *P *< 0.0001; M, *P* = 0.030; A, *P* = 0.0003; P, *P* = 0.0003; MP, *P* = 0.001; AP, *P *< 0.0001. The superscript letters indicate the results of the post hoc comparisons.

Abbreviations: A, agomelatine; AP, agomelatine + progesterone; M, melatonin; MP, melatonin + progesterone; P, progesterone.

### 2.8 Statistical analyses

All data are presented as mean ± standard deviation. Data normality and variance homogeneity were evaluated using the Kolmogorov–Smirnov and Brown–Forsythe tests, respectively. Based on these assumption checks, intergroup comparisons and *post hoc* analyses were performed as follows: parametric data with homogeneous variances were analysed using one‐way ANOVA followed by Tukey's *post hoc* test; parametric data with non‐homogeneous variances were analysed using Welch's ANOVA with Dunnett's T3 *post hoc* test; and non‐parametric data were analysed using the Kruskal–Wallis test followed by Dunn's *post hoc* test. To evaluate pairwise comparisons, Cohen's *d* was utilized for parametric variables, whereas Rosenthal's *r* was calculated for non‐parametric variables. Effect sizes (*d* and *r*) are reported as absolute values. The primary outcome of this study was defined as complete fetus score, while other histological, biochemical and behavioural data were considered secondary outcomes. In all analyses, *P*‐values <0.05 were considered statistically significant. All analyses were conducted using GraphPad Prism 11.0.2 software (GraphPad Software, Boston, MA, USA).

## 3 RESULTS

### 3.1 Confirmation of the experimental abortion model

Mifepristone was administered to all animals except for the control and sham groups on gestational day 11. Within 24 h following mifepristone administration, significant weight loss accompanied by vaginal bleeding was observed. Thus, the successful establishment of the abortion model was demonstrated (Camilleri & Sammut, 2023).

### 3.2 Forced swimming test

Swimming time was significantly longer in the AP group compared to the abortion group (*P* = 0.004, *r* = 0.85) (Figure 2a). In contrast, no significant differences were observed between abortion group and M (*P* = 0.999, *r* = 0.39), A (*P* = 0.231, *r* = 0.59), P (*P* = 0.999, *r* = 0.34) and MP (*P* = 0.999, *r* = 0.34) groups.

**FIGURE 2:**
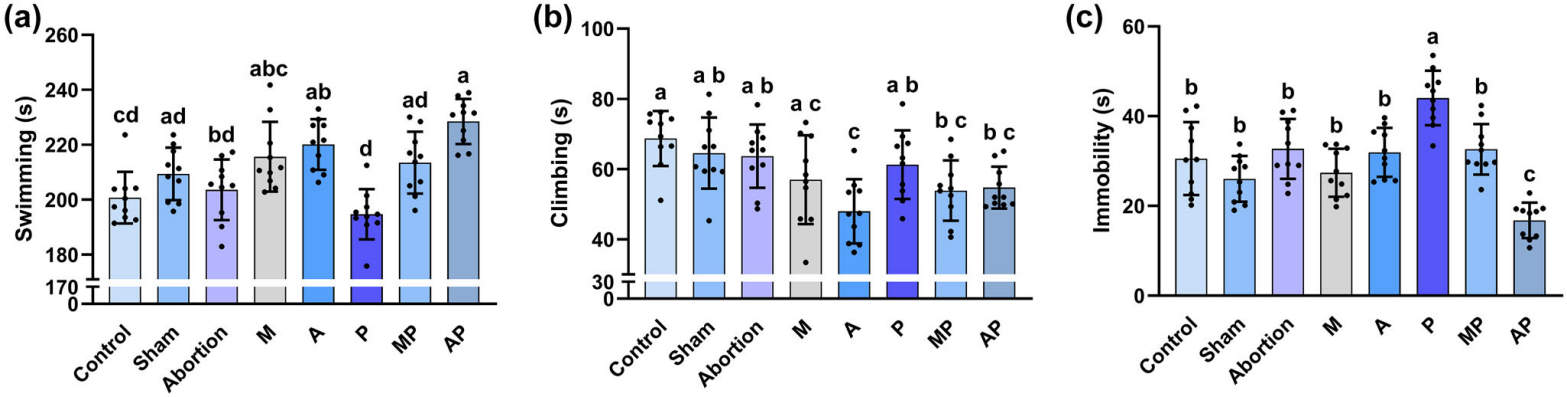
FST swimming time (a), climbing time (b), immobility time (c) of the groups. Data are presented as means ± standard deviation (*n* = 10 per group). Statistical analyses were performed using one‐way ANOVA followed by Tukey's *post hoc* test, or the Kruskal–Wallis test followed by Dunn's *post hoc* test, where appropriate. Groups sharing at least one common letter do not differ significantly, whereas groups not sharing a common letter are statistically significantly different: swimming time (a) compared to abortion group: AP, *P* = 0.004; climbing time (b) compared to abortion group: A, *P* = 0.008; immobility time (c) compared to abortion group: P, *P* = 0.001; AP, *P *< 0.0001. A, agomelatine; AP, agomelatine + progesterone; M, melatonin; MP, melatonin + progesterone; P, progesterone.

Climbing time was significantly shorter in the A group compared to the abortion group (*P* = 0.008, *d* = 1.73) (Figure 2b). However, no significant differences were observed between abortion group and M (*P* = 0.744, *d* = 0.61), P (*P* = 0.999, *d* = 0.26), MP (*P* = 0.281, *d* = 1.11) and AP (*P* = 0.395, *d =* 1.17) groups.

Immobility time was found to be significantly longer in the P group (*P* = 0.001, *d* = 1.78) and significantly shorter in the AP group (*P *< 0.0001, *d* = 2.91) compared to the abortion group (Figure 2c). Conversely, no significant differences were observed between abortion group and M (*P* = 0.474, *d =* 0.88), A (*P* = 0.999, *d =* 0.13) and MP (*P* = 0.999, *d =* 0.02) groups.

### 3.3 Serum hormone analyses results

Serum PGF_2α_ levels were found to be significantly lower in the AP group compared to the abortion group (*P* = 0.004, *d =* 2.48) (Figure 3a).

**FIGURE 3:**
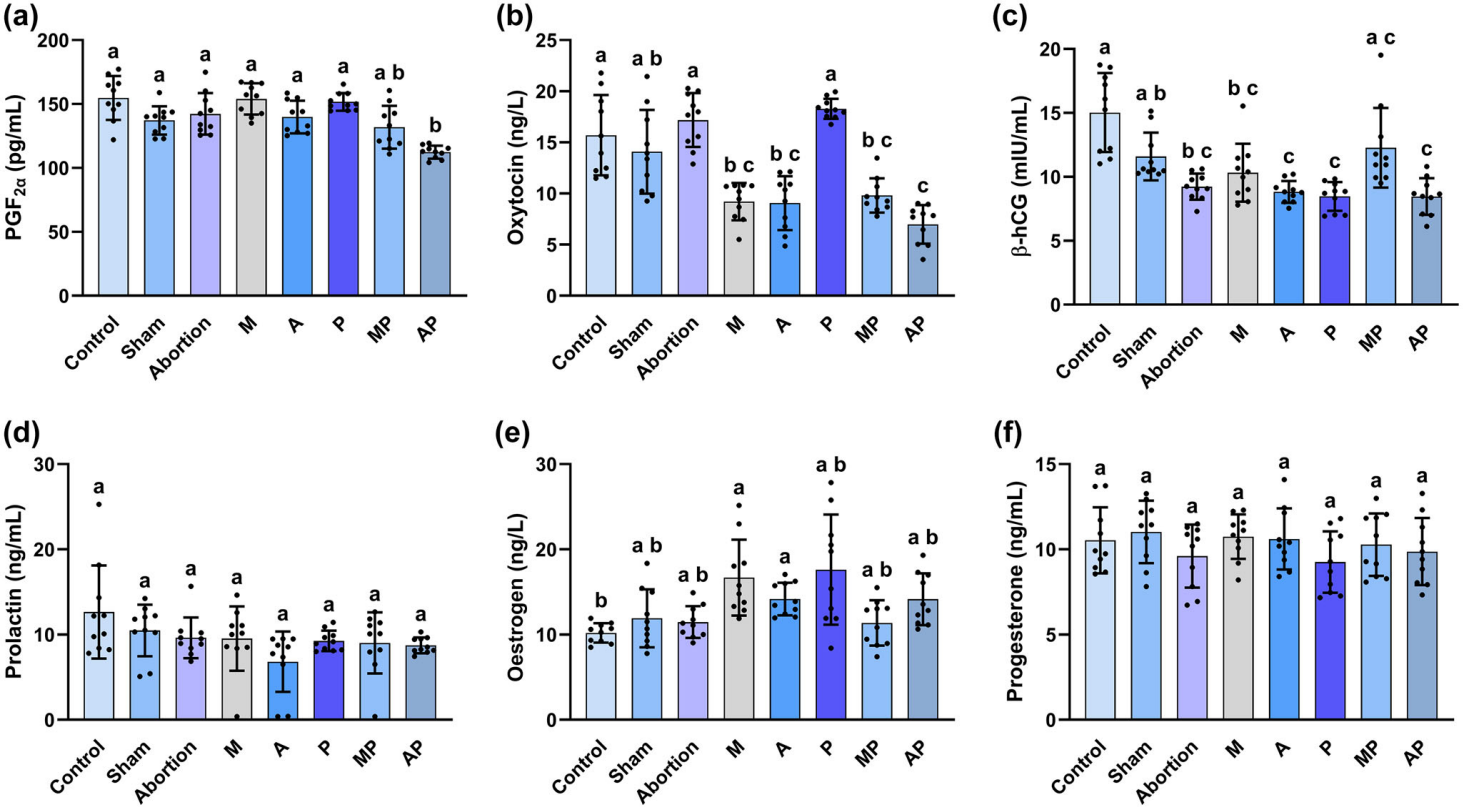
Serum PGF_2α_ levels (a), serum oxytocin levels (b), serum β‐hCG levels (c), serum prolactin levels (d), serum oestrogen levels (e), and serum progesterone levels (f) of the groups. Data are presented as means ± standard deviation (*n* = 10 per group). Statistical analyses were performed using one‐way ANOVA followed by Tukey's *post hoc* test, Welch ANOVA followed by Dunnett's T3 *post hoc* test, or the Kruskal–Wallis test followed by Dunn's *post hoc* test, where appropriate. Groups sharing at least one common letter do not differ significantly, while groups not sharing a common letter are statistically significantly different: serum PGF_2α_ levels (a) compared to abortion group: AP, *P* = 0.004; serum oxytocin levels (b) compared to abortion group: M, *P *< 0.0001; A, *P *< 0.0001; MP, *P *< 0.0001; AP, *P *< 0.0001. A, agomelatine; AP, agomelatine + progesterone; M, melatonin; MP, melatonin + progesterone; P, progesterone.

Serum oxytocin levels were significantly lower in four groups (M, *P *< 0.0001, *d =* 3.52; A, *P *< 0.0001, *d =* 3.08; MP, *P *< 0.0001, *d =* 3.34; AP, *P *< 0.0001, *d =* 4.44) compared to the abortion group (Figure 3b).

No significant differences were detected between the treatment groups (M, A, P, MP, AP) and the abortion group in terms of serum β‐hCG (M, *P* = 0.971, *d =* 0.63; A, *P* = 0.999, *d =* 0.43; P, *P* = 0.935, *d =* 0.71; MP, *P* = 0.224, *d =* 1.31; AP, *P* = 0.979, *d =* 0.62), prolactin (M, *P* = 0.999, *r* = 0.14; A, *P* = 0.999, *r* = 0.36; P, *P* = 0.999, *r* = 0.05; MP, *P* = 0.999, *r* = 0.05; AP, *P* = 0.999, *r* = 0.21), oestrogen (M, *P* = 0.099, *d =* 1.53; A, *P* = 0.107, *d =* 1.44; P, *P* = 0.251, *d =* 1.29; MP, *P* = 0.999, *d =* 0.04; AP, *P* = 0.461, *d =* 1.07) and progesterone (M, *P* = 0.845, *d =* 0.72; A, *P* = 0.913, *d =* 0.55; P, *P* = 0.999, *d =* 0.19; MP, *P* = 0.990, *d =* 0.37; AP, *P* = 0.999, *d =* 0.14) levels (Figure 3c,d,e,f).

### 3.4 Histological examination

#### 3.4.1 Macroscopic scoring

Images for macroscopic scoring are shown in Figure 4. The abortion group exhibited a significantly lower complete fetus score compared to all other groups (control, *P *< 0.0001, *r* = 1.25; sham, *P *< 0.0001, *r* = 1.16; M, *P* = 0.003, *r* = 0.87; A, *P* = 0.001, *r* = 0.96; P, *P *< 0.0001, *r* = 1.07; MP, *P* = 0.0003, *r* = 0.98; AP, *P *< 0.0001, *r* = 1.16). Conversely, the scores for fetal remains (control, *P *< 0.0001, *r* = 5.55; sham, *P *< 0.0001, *r* = 5.55; M, *P* = 0.006, *r* = 0.83; A, *P *< 0.0001, *r* = 1.04; P, *P* = 0.001, *r* = 0.95; MP, *P *< 0.0001, *r* = 1.06; AP, *P *< 0.0001 *r* = 1.15) and bamboo‐like appearance (control, *P *< 0.0001, *r* = 1.23; sham, *P *< 0.0001, *r* = 1.23; M, *P* = 0.006, *r* = 0.83; A, *P* = 0.001, *r* = 0.92; P, *P* = 0.001, *r* = 0.95; MP, *P *< 0.0001, *r* = 1.05; AP, *P *< 0.0001, *r* = 1.15) were found to be significantly higher in the abortion group than in all other groups (Table 1).

**FIGURE 4:**
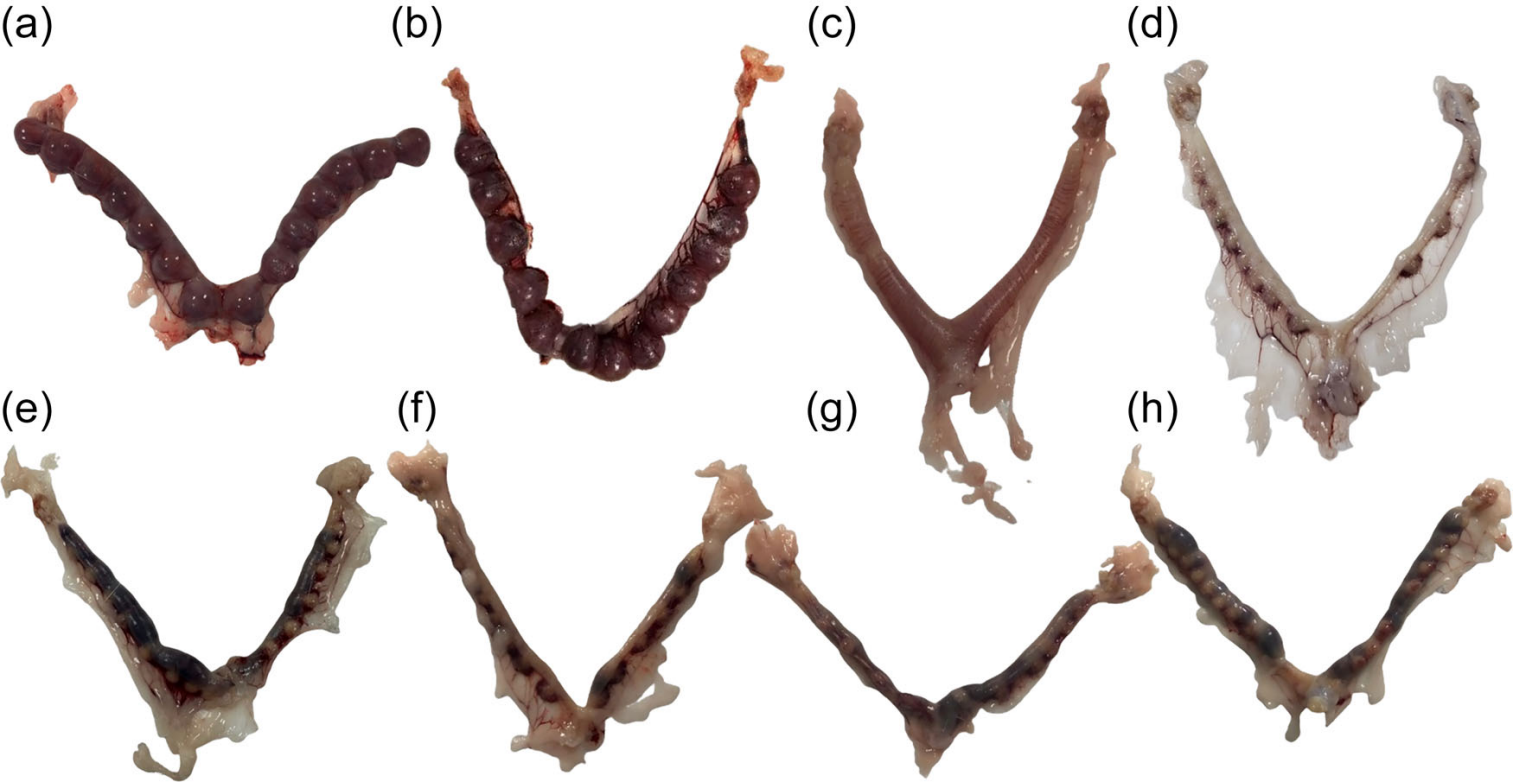
Macroscopic images of uterine tissues (a: control; b: sham; c: abortion; d: melatonin; e: agomelatine; f: progesterone; g: melatonin+progesterone; h: agomelatine + progesterone).

#### 3.4.2 Microscopic scoring

Images for microscopic scoring are shown in Figures 5, 6, 7, 8. Compared to all other groups, scores for decidual congestion (control, *P* = 0.007, *r* = 0.82; sham, *P* = 0.002, *r* = 0.90; M, *P* = 0.0003, *r* = 0.98; A, *P *< 0.0001, *r* = 1.07; P, *P *< 0.0001, *r* = 1.15; MP, *P* = 0.0003, *r* = 0.98; AP, *P* = 0.029, *r* = 0.73), decidual inflammation (control, *P* = 0.0002, *r* = 1.01; sham, *P* = 0.005, *r* = 0.84; M, *P* = 0.005, *r* = 0.92; A, *P* = 0.001, *r* = 0.92; P, *P* = 0.0002, *r* = 1.01; MP, *P *< 0.0001, *r* = 1.09; AP, *P *< 0.0001, *r* = 1.09), cytotrophoblast cells (control, *P *< 0.0001, *r* = 1.07; sham, *P *< 0.0001, *r* = 1.16; M, *P* = 0.009, *r* = 0.81; A, *P *< 0.0001, *r* = 1.07; P, *P* = 0.002, *r* = 0.90; MP, *P *< 0.0001, *r* = 1.07; AP, *P* = 0.0003, *r* = 0.98) and syncytiotrophoblast cells (control, *P *< 0.0001, *r* = 1.17; sham, *P *< 0.0001, *r* = 1.08; M, *P* = 0.030, *r* = 0.73; A, *P* = 0.0003, *r* = 0.99; P, *P* = 0.0003, *r* = 0.99; MP, *P* = 0.001, *r* = 0.91; AP, *P *< 0.0001, *r* = 1.08) were found to be significantly lower in the abortion group (Table 2). Additionally, the necrosis score was significantly higher in the abortion group compared to both the control (*P* = 0.011, *r* = 0.79) and sham groups (*P* = 0.030, *r* = 0.73) (Table 2).

**FIGURE 5:**
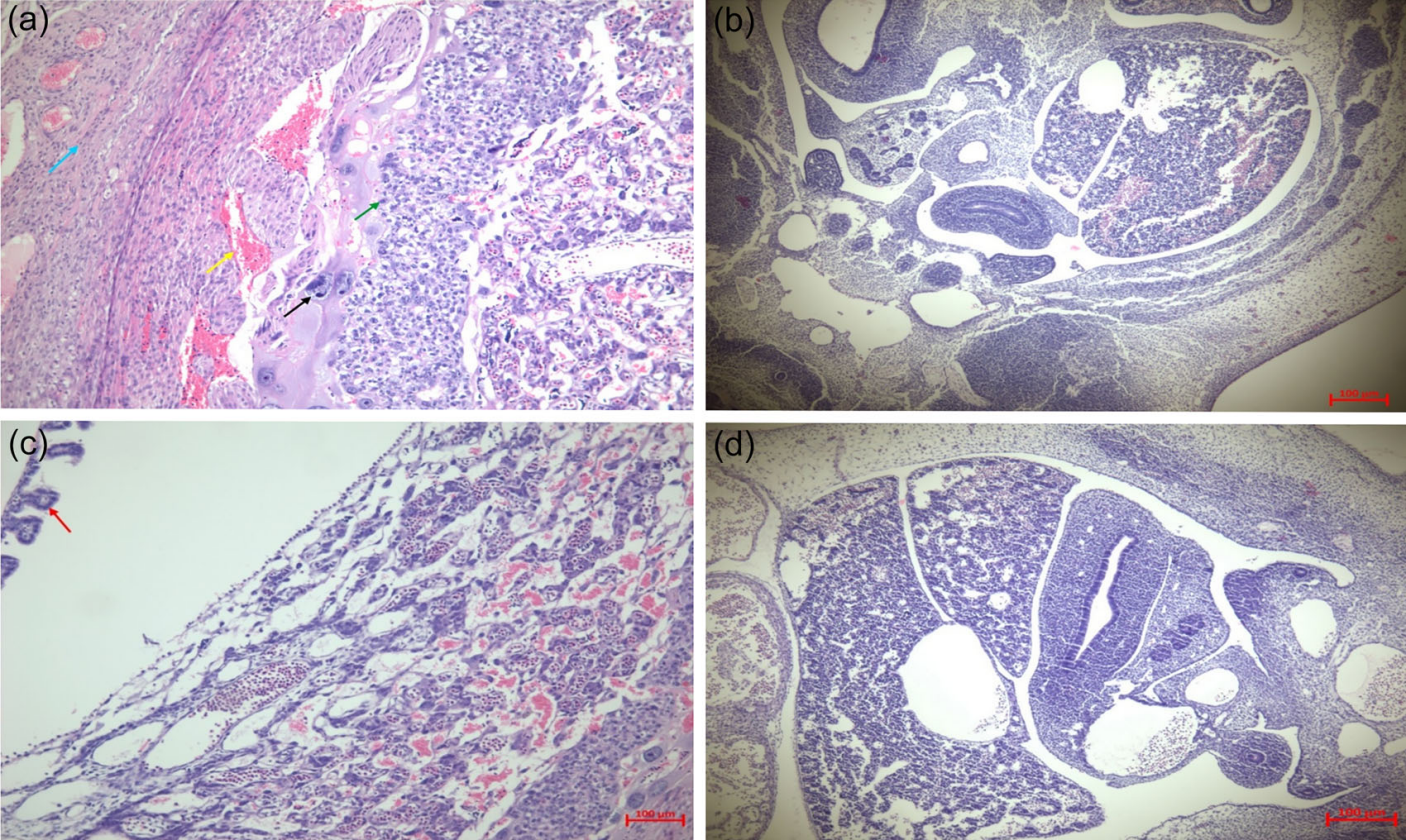
Uterine sections and immature fetuses in the control and sham groups. (a) Uterine section of the control group. Yellow arrow, haemorrhagic areas; green arrow, cytotrophoblasts; black arrow, syncytiotrophoblasts; light blue arrow, inflammatory cells. (b) Immature fetus in the control group. (c) Uterine section of the sham group. Red arrow: chorionic epithelium. (d) Immature fetus in the sham group.

**FIGURE 6:**
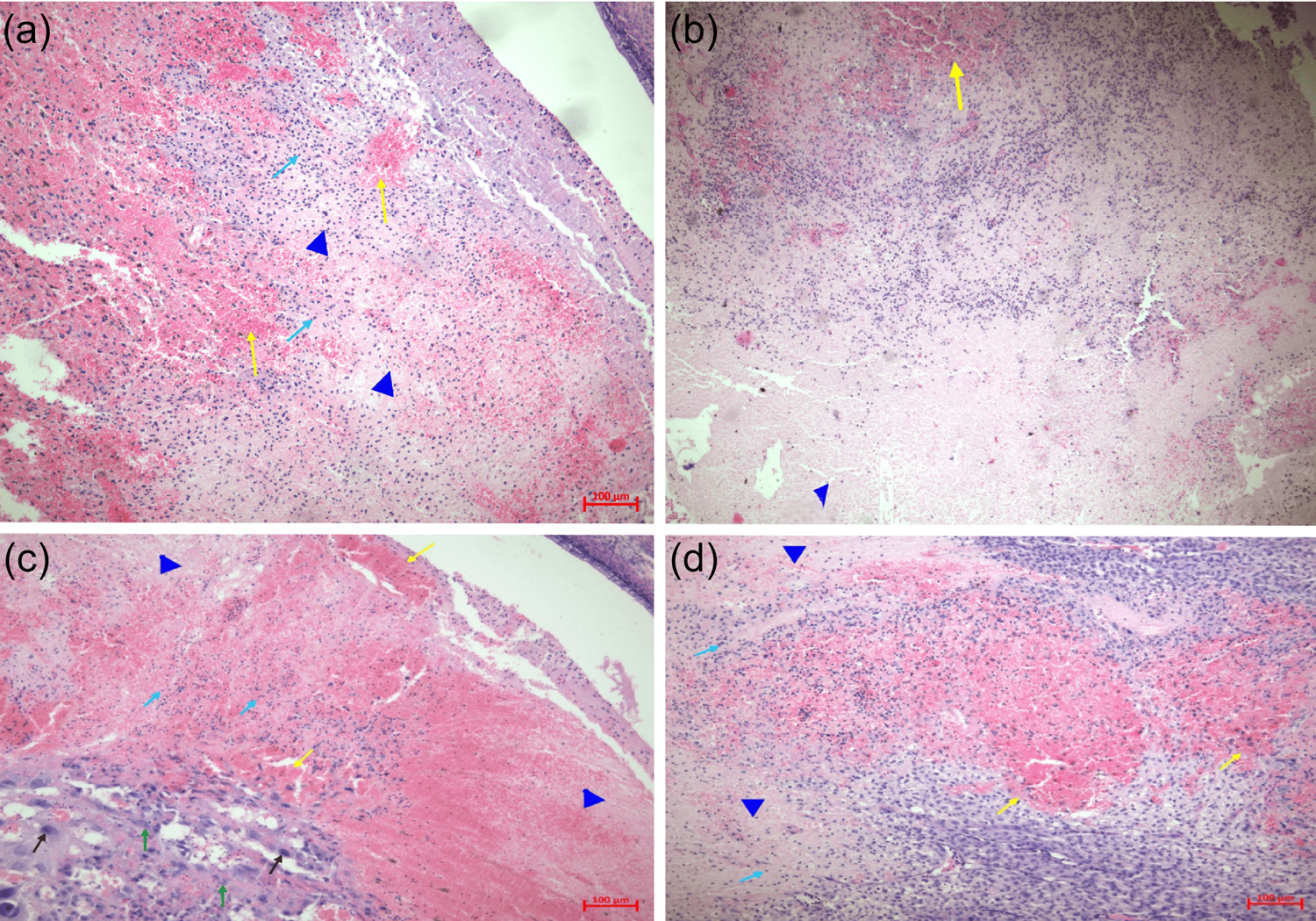
Uterine sections of the abortion and melatonin groups. (a, b) Uterine section of the abortion group. (c, d) Uterine section of the melatonin group. Dark blue triangle, necrotic decidua; yellow arrow, haemorrhagic areas; green arrow, cytotrophoblasts; black arrow, syncytiotrophoblasts; light blue arrow, inflammatory cells.

**FIGURE 7:**
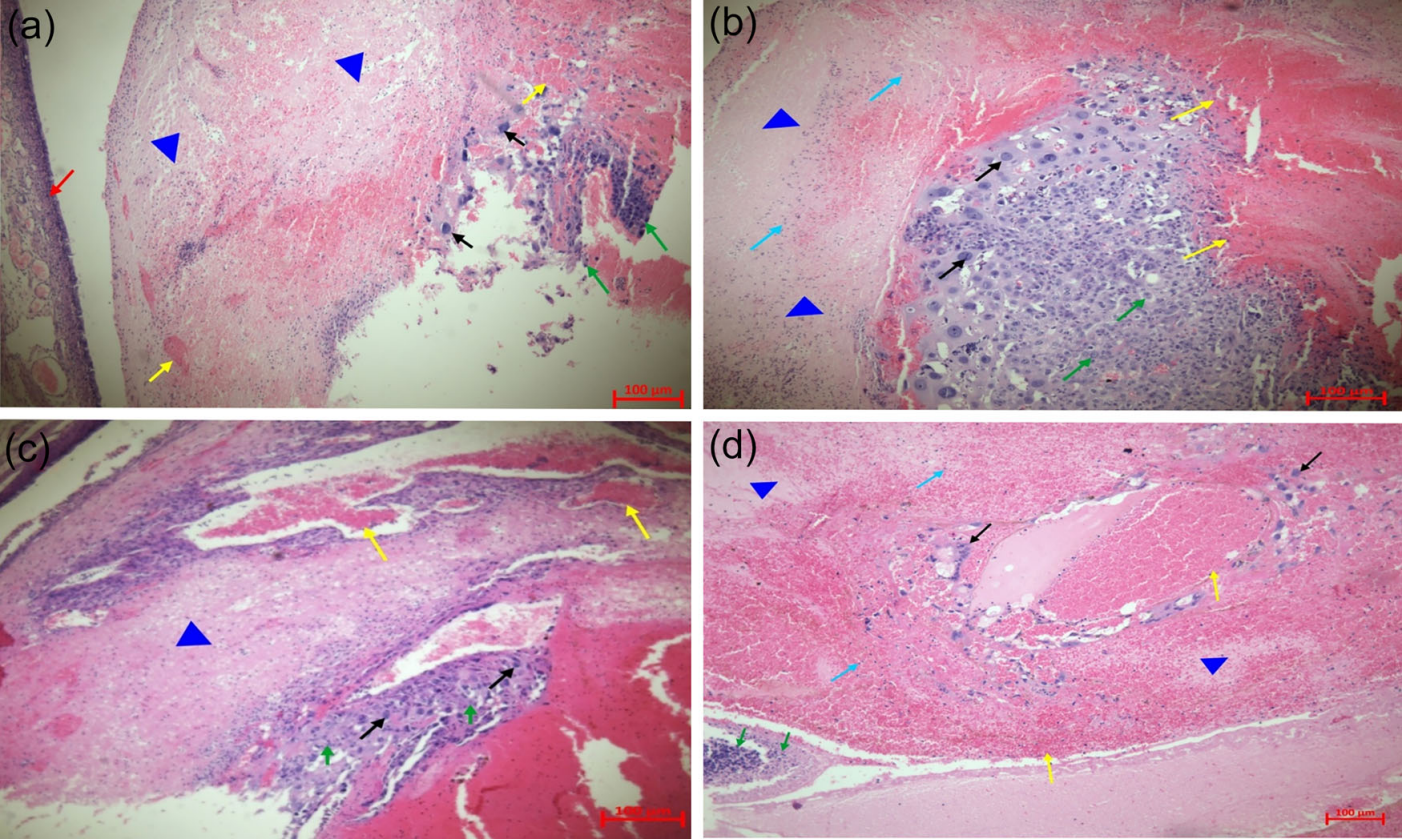
Uterine sections of the agomelatine and progesterone groups. (a, b) Uterine section of the agomelatine group. (c, d) Uterine section of the progesterone group. Dark blue triangle, necrotic decidua; yellow arrow, haemorrhagic areas; green arrow, cytotrophoblasts; black arrow, syncytiotrophoblasts; light blue arrow, inflammatory cells; red arrow, chorionic epithelium.

**FIGURE 8:**
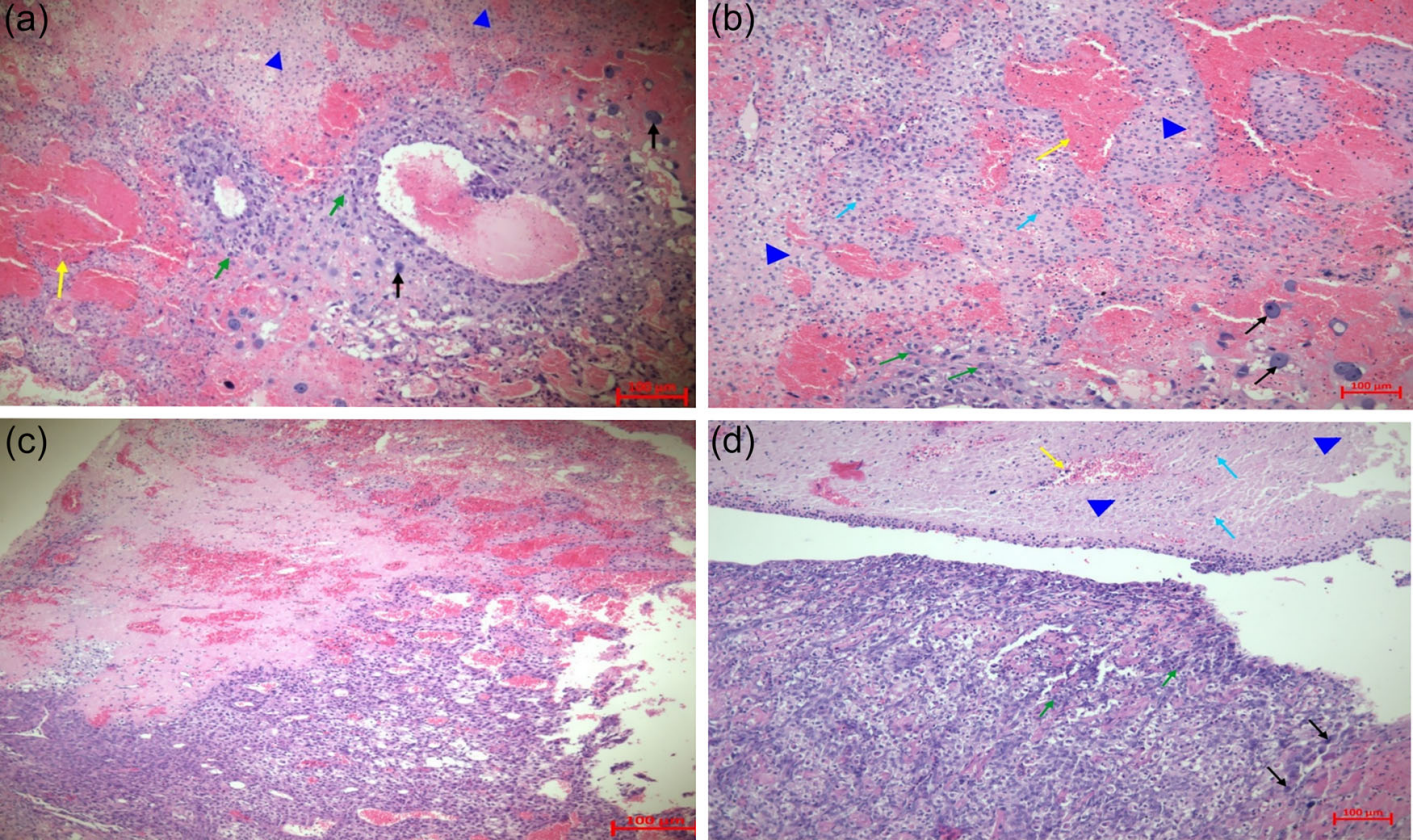
Uterine section of the melatonin + progesterone and agomelatine + progesterone groups. (a, b) Uterine section of the melatonin + progesterone group. (c, d) Uterine section of the agomelatine + progesterone group. Dark blue triangle: necrotic decidua; yellow arrow, haemorrhagic areas; green arrow, cytotrophoblasts; black arrow, syncytiotrophoblasts; light blue arrow, inflammatory cells.

## 4 DISCUSSION

Our findings demonstrate that agomelatine exerts various beneficial effects on behavioural, biochemical and histological parameters in an experimental model of abortion.

The forced swimming test (FST) was conducted to evaluate the efficacy of progesterone, melatonin and agomelatine on stress and depressive‐like behaviours – key factors in the aetiology of abortion – within the experimental abortion model. When comparing active behaviours, swimming duration – considered a positive behavioural marker in the FST – was significantly longer following the combined administration of agomelatine and progesterone compared to the abortion group, indicating a positive response against depressive‐like behaviour (Figure 2a). Conversely, climbing duration, another active parameter associated with survival effort, was shorter following agomelatine administration relative to the abortion group (Figure 2b). Rather than a loss of overall mobility, this finding should be evaluated considering that animals may have engaged in swimming behaviour when not climbing. Therefore, immobility duration provides a clearer analysis for assessing depressive‐like behaviour in FST protocols (Ghazizadeh et al., 2020). The immobility period, considered an indicator of depressive‐like behaviour, was longer following progesterone monotherapy compared to that observed in the abortion group (Figure 2c). In alignment with a pharmacovigilance study suggesting that progesterone‐derivative treatments may induce depressive‐like behaviours, the current findings indicate that progesterone increases depressive behaviour (Gao et al., 2025). When assessing immobility duration across the various treatment regimens, a significant reduction compared to the abortion group was observed exclusively following the combined administration of agomelatine and progesterone (Figure 2c). These data demonstrate that the depressive‐like effects induced by progesterone were reversed by agomelatine. Furthermore, while the immobility period was significantly shorter following the combined administration of agomelatine and progesterone compared to that observed in the abortion group, no such difference was observed following the combined administration of melatonin and progesterone; this suggests that agomelatine is more effective than melatonin in improving progesterone‐induced depressive‐like behaviours when used in combination therapy. This outcome can be explained by agomelatine's antidepressant effect (Su et al., 2023). In this context, the inclusion of melatonin groups provided a baseline for comparison to evaluate whether the observed effects of agomelatine stem solely from melatonergic actions or whether drug‐specific, non‐melatonergic effects also contribute to the outcome. Various studies in the literature have reported that agomelatine administration shortens the immobility duration in FST protocols (Bourin et al., 2004; Ladurelle et al., 2012). The absence of a significant difference in immobility duration in the group receiving agomelatine alone in the current study may stem from differences in factors such as animal species, drug dosage and duration of administration across studies. However, the significant reduction of immobility time following the combined administration of agomelatine and progesterone appears consistent with the antidepressant effects of agomelatine reported in the literature. Considering a meta‐analysis stating that psychological stress can increase the risk of abortion by up to 42%, the clinical use of progesterone – the single treatment option which may trigger depressive‐like behaviour – could further complicate the management of abortion (Qu et al., 2017). Among the primary objectives of the current study was to evaluate the psychological and behavioural effects of progesterone – the current standard treatment for abortion – alongside those of novel agents. Given that maternal stress is an established risk factor that compromises pregnancy maintenance, achieving behavioural integrity while preserving gestation physiology represents a critical therapeutic target that may not be completely covered by standard progesterone monotherapy. Based on our findings, agomelatine may offer superior potential compared to both progesterone monotherapy and melatonin administration, particularly in pregnancies where depressive factors such as stress and anxiety are predominant. These findings, alongside further research, offer a novel perspective on the highly limited therapeutic options for abortion.

PGF_2α_ levels were found to be significantly lower only following the combined administration of agomelatine and progesterone compared to the abortion group (Figure 3a). Considering that elevated PGF_2α_ levels increase myometrial contractions, which poses a potential risk for abortion, the inhibitory effect of combined agomelatine and progesterone administration on PGF_2α_ is a protective finding regarding abortion (Li et al., 2021). Furthermore, it is well established that high levels of progesterone during gestation reduce uterine contractions by inhibiting PGF_2α_ synthesis (Farina et al., 2004). Similarly, agomelatine has been shown in the literature to inhibit PGF_2α_‐induced contractions in the rat myometrium (Kutlu et al., 2015). The combined administration of progesterone and agomelatine – both of which have been previously shown in the literature to be effective on PGF_2α_ levels – appears to be more effective on PGF_2α_ levels compared to the respective monotherapies of melatonin, agomelatine or progesterone, as well as the combined administration of melatonin and progesterone in the present study.

Oxytocin levels were found to be significantly lower following the monotherapy or combined administration of melatonin and agomelatine with progesterone compared to those of the abortion group (Figure 3b). It has been reported in the literature that oxytocin, a uterotonic hormone, can trigger abortion through increased receptor expression during the early stages of gestation (Liu et al., 2020). Therefore, it is suggested that the decrease in oxytocin levels may exert a risk‐reducing effect on abortion by inhibiting myometrial contractions. Melatonin has been shown to inhibit oxytocin release in rats via MT_1_ receptor signalling (Juszczak et al., 2016). In line with these findings, our previous study in rats demonstrated that chronic agomelatine administration reduced oxytocin levels (Kacar et al., 2023). In the present study, the lower oxytocin levels observed following the monotherapy or combined administration of melatonin and agomelatine with progesterone compared to those of the abortion group is consistent with the literature; furthermore, the lack of a significant difference between the melatonin‐ and agomelatine‐based treatments suggests that this effect is mediated through melatonergic mechanisms. While further studies are required to clarify the underlying processes, these effects of melatonin and agomelatine on oxytocin levels are protective regarding the pathophysiology of abortion.

In the present study, no significant differences were detected in β‐hCG, prolactin, oestrogen and progesterone levels when the abortion group was compared to the monotherapies of melatonin, agomelatine or progesterone, as well as the combined administrations of melatonin or agomelatine with progesterone (Figure 3c,d,e,f). These data suggest that the protective effects of melatonin and agomelatine during gestation might be mediated through molecular mechanisms other than primary hormonal signalling pathways, such as anti‐inflammatory or antioxidant pathways (Tamura et al., 2020). However, one of the limitations of our study is that these specific mechanisms were not investigated. Furthermore, the fact that prolactin levels in rodents remain low and stable from approximately the 10th day of gestation until birth may explain the lack of difference in the values measured on the 14th day, which was the sacrifice day of the current study (Grattan et al., 2008). Even if progesterone and oestrogen levels in the blood do not change, their receptor expression or sensitivity at the tissue level may have altered; therefore, receptor determination in future studies will be beneficial (Manvelyan et al., 2022). Additionally, in the current study, serum samples were not collected from the animals prior to the sacrifice day to avoid interfering with behavioural testing; however, obtaining serum samples on different days of gestation in subsequent studies could provide a better understanding of the fluctuations in hormone levels.

The observation of viable fetuses and the high complete fetus scores in the control and sham groups confirm the preservation of the physiological course of gestation. Conversely, the presence of a bamboo‐like appearance in the uterus instead of complete fetuses in the abortion group demonstrates the successful establishment of the experimental abortion model (Table 1, Figure 4). Compared to the abortion group, the increase in complete fetus scores following the administration of melatonin, agomelatine and progesterone – either as monotherapies or in combination with progesterone – reaching levels similar to the control group, and the concurrent significant decrease in bamboo‐like appearance scores indicate that these therapeutic agents exert a distinct gestation‐protective effect (Table 1). This finding is consistent with literature reporting that progesterone supports endometrial integrity and the maintenance of gestation (DeMayo & Lydon, 2020). Furthermore, the high complete fetus scores obtained following the monotherapy or combined administration of melatonin and agomelatine with progesterone, which were similar to those of the control group, are consistent with the positive effects of melatonin and agomelatine on reproductive physiology previously reported in the literature (Tamura et al., 2008; Zhao et al., 2024).

The healthy maintenance of fetal development depends on the formation and preservation of an appropriate intrauterine microenvironment. Cytotrophoblast and syncytiotrophoblast cells, which are fundamental elements of this microenvironment, are responsible for maintaining the structural integrity of the placental barrier as well as overseeing endocrine functions and fetal oxygenation (Costa, 2016). Another critical factor for maintaining optimum conditions is the presence of adequate decidual congestion (Norwitz et al., 2001). In cases of insufficient congestion, placental development is impaired and the risk of abortion increases (Tong et al., 2022). In light of this information, the finding that both decidual congestion and cytotrophoblast–syncytiotrophoblast cell scores were lower in the abortion group compared to the control group in the present study is consistent with the literature (Table 2). The observation that decidual congestion and cytotrophoblast–syncytiotrophoblast cell scores following the administration of melatonin, agomelatine and progesterone – either as monotherapies or in combination with progesterone – were higher than those in the abortion group and similar to the control group demonstrates that these administered agents exert protective effects in the maintenance of gestation (Table 2). It is reported in the literature that progesterone supports placental development during gestation by promoting the maturation of cytotrophoblasts into syncytiotrophoblasts, thereby preserving the integrity and functionality of the placental barrier (Burton & Jauniaux, 2023). Additionally, progesterone contributes to the maintenance of decidual congestion within physiological limits by modulating uterine vascular tone (Goddard et al., 2014). Furthermore, melatonin is reported to have protective and regulatory effects on pregnancy physiology. Among these effects, supporting decidualization and regulating uteroplacental blood flow hold significant importance (Cui et al., 2021). Melatonin also prevents cell loss by reducing oxidative stress‐induced apoptosis in villous cytotrophoblasts, thus contributing to the preservation of placental integrity (Lanoix et al., 2013). Moreover, it plays a role in the renewal and functional continuity of the syncytiotrophoblast layer by supporting the differentiation of cytotrophoblasts into syncytiotrophoblasts (Verteramo et al., 2022). A previous study reported that specific polymorphisms in the melatonin receptor gene (*MTNR1B*) can adversely affect the process of embryo attachment to the endometrium, leading to recurrent implantation failure and early pregnancy loss (Robeva et al., 2023). Although the effects of melatonin in pregnancy have been investigated, data regarding agomelatine remain limited. In this context, our previous study demonstrated that chronic agomelatine administration prolonged gestation and increased the number of fetuses (Kacar et al., 2023). In the present study, the fact that decidual congestion and cytotrophoblast–syncytiotrophoblast cell scores in the agomelatin‐treated groups were similarly high as in the melatonin group indicates that the drug exhibits a significant protective effect at the tissue level in maintaining pregnancy, similar to melatonin. Furthermore, the fact that agomelatine has an antidepressant profile, unlike melatonin, suggests that it may offer an additional potential therapeutic advantage against stress factors that play a critical role in the aetiopathogenesis of pregnancy loss. However, further mechanistic studies are needed to clarify the specific molecular pathways behind these observed effects.

The literature emphasizes that physiological inflammatory responses are essential for the maternal immune acceptance of the semi‐allogeneic fetus and the healthy progression of pregnancy, playing a supportive role in maternal–fetal interactions (Negishi & Morita, 2024; Yang et al., 2019). In the present study, while the decidual inflammation score was observed to be lower in the abortion group compared to the control group, these scores were found to be as high as those of the control group following the administration of melatonin, agomelatine and progesterone, whether as monotherapies or in combination with progesterone (Table 2). The termination of the experiment 3 days following the induction of the abortion model is a possible factor that could explain the low decidual inflammation score detected in the abortion group, as it may have resulted in the complete expulsion of fetal and decidual tissues (Table 2). On the other hand, the lack of a significant difference between the treatment groups and the control group indicates that the observed inflammatory response followed a course within the physiological limits specific to the gestation process. This finding demonstrates that the administered agents do not interfere with the physiological adaptation processes of pregnancy and supports their reliability for use during gestation.

The significantly higher necrosis score in the abortion group compared to the control group – a parameter indicating the disruption of decidual integrity – confirms the successful establishment of the model (Table 2). The fact that the significant difference existing between the control group and the abortion group was eliminated when comparing the control group with the monotherapies of melatonin, agomelatine or progesterone, as well as the combined administrations of melatonin or agomelatine with progesterone, suggests that these administered agents have a limiting effect on the development of decidual necrosis in a gestation‐protective manner (Table 2). In the present study, the finding that necrosis scores following the monotherapy or combined administration of melatonin and agomelatine with progesterone showed no statistical difference from those of the control group reveals that both agents can exert a contribution to the maintenance of pregnancy similar to progesterone, whose role in supporting decidual integrity is well recognized (Bhurke et al., 2016).

This study has several limitations that warrant consideration. First, the administered agents were applied at a single pharmacological dose during the light phase, which limits dose–response evaluations and phase‐controlled diurnal efficacy assessments. Second, as an initial study, initiating treatment on gestational day 0 in our experimental procedure aimed both to evaluate the effects of the administered agents starting specifically from early gestation and to assess the potential of a protective approach for high‐risk conditions such as recurrent pregnancy loss. Inducing the abortion model 3 days prior to sacrifice was specifically designed to capture acute biochemical alterations and early histopathological responses before endogenous tissue‐repair mechanisms could mask these outcomes. However, this framework does not fully reflect clinical scenarios where therapeutic intervention begins only after diagnosis, nor does it evaluate long‐term maternal and fetal outcomes. Third, tissue‐level receptor expression assays and specific inflammatory/oxidative stress parameters were not measured, rendering the cellular‐level evidence indirect. Furthermore, while the absence of hormone tracking across different time points was a necessary methodological choice to avoid procedural stress prior to behavioural testing, it limits a comprehensive characterization of the hormonal profile. To address these boundaries, future mechanistic studies combining phase‐controlled multi‐dose paradigms, receptor‐specific evaluations, inflammatory/oxidative stress parameters and post‐diagnostic protocols are required.

### 4.1 Conclusion

The ability of agomelatine to ameliorate depressive‐like behaviours in an experimental abortion model is of great significance, considering that stress and depression are critical risk factors for pregnancy loss. From a hormonal perspective, the combined use of agomelatine with progesterone was found to exert a protective effect on pregnancy maintenance by reducing serum PGF_2α_ and oxytocin levels. Histological analyses demonstrated that treatments involving melatonin and agomelatine increased the complete fetus score and supported the continuity of the intrauterine microenvironment by preserving decidual congestion levels, the integrity of cytotrophoblast–syncytiotrophoblast cells and physiological inflammatory responses. Furthermore, both agents suggested a protective effect against decidual necrosis. The therapeutic effects of agomelatine offer a novel perspective on the currently limited treatment options for abortion and are poised to guide further research in this field.

## AUTHOR CONTRIBUTIONS

The experimental procedures of this study were conducted at the Firat University Experimental Research Center. Mehtap Pervin: Data curation, formal analysis, investigation, visualization, writing–original draft, writing–review & editing. Zeynep Dila Oz: Conceptualization, investigation, validation, writing–review & editing. Ahmed Sait Bozyil: Data curation, investigation, writing–review & editing. Orhan Sayin: Data curation, formal analysis, investigation, writing–review & editing. Husna Batmaz: Data curation, investigation, visualization, writing–review & editing. Serhat Hancer: Data curation, investigation, writing–review & editing. Salih Burcin Kavak: Conceptualization, methodology, writing–review & editing. Nevin Kocaman: Conceptualization, data curation, writing–review & editing. Haluk Kelestimur: Conceptualization, methodology, writing–review & editing. Emine Kacar: Conceptualization, data curation, formal analysis, methodology, project administration, supervision, validation, writing–review & editing. All authors have read and approved the final version of this manuscript and agree to be accountable for all aspects of the work in ensuring that questions related to the accuracy or integrity of any part of the work are appropriately investigated and resolved. All persons designated as authors qualify for authorship, and all those who qualify for authorship are listed.

## CONFLICT OF INTEREST

None declared.

## GENERATIVE AI STATEMENT

Google Gemini (Gemini 1.5 Pro) was utilized solely for language editing and grammatical refinement of this manuscript. All scientific content, data interpretation and final conclusions were authored and verified entirely by the authors, who assume full responsibility for the work.

## Data Availability

The data that support the findings of this study are available from the corresponding author upon reasonable request.
